# Artificial Intelligence in Medicine With Emphasis on Orthopedic Practice

**DOI:** 10.7759/cureus.98306

**Published:** 2025-12-02

**Authors:** Jakub Bartkowski, Julia Zerdka, Patryk Brasse, Mateusz Piszka, Eliza Kwapien, Karolina Staszkiewicz, Maria Kubicka, Kacper K Staszkiewicz, Filip Czarnecki

**Affiliations:** 1 Medicine, Miejskie Zakłady Opieki Zdrowotnej w Żorach, Żory, POL; 2 Medicine, Wojewódzki Szpital Specjalistyczny nr 5 im. św. Barbary, Sosnowiec, POL; 3 Medicine, Samodzielny Publiczny Szpital Kliniczny im. Andrzeja Mielęckiego w Katowicach, Katowice, POL; 4 Orthopedics, Samodzielny Publiczny Zakład Opieki Zdrowotnej Ministerstwa Spraw Wewnętrznych i Administracji (SP ZOZ MSWiA), Katowice, POL; 5 Medicine, Szpital Miejski w Siemianowicach Śląskich, Siemianowice Śląskie, POL

**Keywords:** ai-driven diagnostics, artificial intelligence in medicine, medical education and training, medical interview, orthopedic surgery

## Abstract

This paper presents a comprehensive review of the impact of artificial intelligence (AI) on healthcare, with a particular focus on its applications in orthopedic medicine. AI technologies, notably large language models (LLMs) and machine learning (ML) algorithms, have increasingly enhanced diagnostic accuracy, clinical decision-making, and personalized patient care. The integration of AI into medical education and training fosters improved learning outcomes through interactive simulations and tailored curricula. In orthopedics, AI-driven imaging tools employing deep learning techniques demonstrate superior performance in fracture detection, cartilage segmentation, and musculoskeletal injury diagnosis, often rivaling expert human clinicians. Moreover, AI models show promise in automating medical history taking and supporting diagnostic workflows, although real-world implementation remains limited by data quality, system integration, and ethical concerns. The evaluation of LLMs such as ChatGPT in standardized medical examinations reveals both their potential and current limitations in clinical reasoning. Ethical and technical challenges, including data bias, accountability, privacy, and the need for transparency, emerge as critical considerations for safe AI adoption in clinical practice. The future of AI in orthopedics lies in multidisciplinary collaboration, robust validation, and the development of multimodal models that integrate diverse biomedical data, paving the way for precision medicine and enhanced patient outcomes.

## Introduction and background

Artificial Intelligence (AI) has evolved rapidly from a specialized discipline of computer science into an integral component of modern society. AI technologies enhance daily life, stimulate academic inquiry, and increasingly permeate various sectors of the economy, including healthcare. Within this domain, AI is transforming clinical practice by improving the accuracy, efficiency, and personalization of medical services. Through the application of advanced algorithms and machine learning (ML) techniques, AI facilitates the analysis of vast and complex clinical datasets, thereby supporting more accurate disease diagnosis, optimized treatment planning, and continuous patient monitoring. Its applications encompass early disease detection, such as in cancer and sepsis, drug discovery acceleration, and the streamlining of administrative workflows. As AI systems become progressively embedded in healthcare infrastructure, they hold the potential to revolutionize patient care delivery, reduce healthcare expenditures, and advance precision medicine. However, their implementation concurrently raises critical ethical, legal, and regulatory concerns that must be addressed to ensure safe and equitable deployment. AI has also become a catalyst for interdisciplinary collaboration, integrating medical expertise with computational, ethical, and policy-driven perspectives. This convergence enables the production of actionable insights that enhance public health outcomes. Furthermore, the incorporation of AI into telemedicine, wearable technologies, and population health analytics has significantly increased the accessibility and continuity of care, particularly in underserved regions. By automating routine diagnostic tasks, AI allows clinicians to allocate more time to patient-centered decision-making, thereby enhancing empathy and human connection in an increasingly digital healthcare environment. Beyond operational efficiency, AI serves as a cornerstone of evidence-based medicine by enabling continuous learning from real-world clinical data and by supporting predictive models that can anticipate disease progression before symptoms manifest. Advanced AI systems are now being integrated into population-level surveillance networks to detect epidemiological trends, antimicrobial resistance, and emerging infectious threats, thereby enhancing global health security. As regulatory bodies and international organizations begin to emphasize algorithmic accountability and transparency, the focus has shifted from mere technological adoption to the creation of ethical frameworks that align AI’s capabilities with societal and humanitarian values.

Among the most significant AI tools applicable to professional medical practice are large language models (LLMs) and ML-based systems. LLMs represent advanced forms of AI capable of understanding and generating human language. Trained on a vast text data from diverse sources, both print and online, LLMs learn linguistic patterns encompassing context, semantics, syntax, and style [1]. Their precision and utility are determined by the quality and relevance of the underlying training data. To maximize domain applicability, LLMs should be trained on field-specific corpora [2]. Empirical evidence indicates that domain-adapted biomedical models, such as BioBERT, ClinicalBERT, and BioMedRoBERTa, demonstrate superior performance in biomedical text recognition compared with general-purpose language models [3,4]. Emerging research further indicates that hybrid systems combining LLMs with multimodal inputs, such as medical imaging, laboratory, and genomic data, can enhance diagnostic reasoning and clinical decision support. These capabilities suggest a future in which LLMs not only assist in documentation and communication but also act as intelligent intermediaries that interpret and contextualize patient data in real time. ML, as a core subfield of AI, focuses on developing computational algorithms that enable systems to identify patterns and infer outcomes based on empirical data [5,6]. The introduction of ML-driven tools has numerous applications across medical education, diagnostics, and therapeutic management, underscoring AI’s pivotal role in the evolution of contemporary healthcare [7-10]. A prominent example is OpenEvidence, an AI-powered clinical decision support platform that integrates natural language processing to provide rapid, evidence-based, fully referenced answers drawn from over 35 million peer-reviewed publications and leading medical journals. Access is restricted to verified healthcare professionals, ensuring reliability and security. It functions not only as a powerful medical literature search engine but also synthesizes complex information into concise, actionable clinical guidance, supporting physician decision-making at the point of care without supplanting clinical judgment. OpenEvidence’s advanced AI capabilities include refining clinical notes, conducting real-time literature reviews, and generating research briefs, thereby streamlining clinical workflows, enhancing diagnostic accuracy, and promoting evidence-based practice. By combining transparency, speed, and comprehensive sourcing, OpenEvidence exemplifies the transformative potential of AI to bridge scientific knowledge and clinical application in modern healthcare environments.

## Review

Methodology

This article is a narrative review of the literature aimed at gathering and organizing current scientific evidence on the applications and development of AI in medicine, with particular emphasis on orthopedic surgery.

The literature search was conducted in the PubMed and Google Scholar databases using keywords such as "artificial intelligence", "AI-driven imaging", "orthopedic surgery", "fracture detection", "history taking", "large language model", and "machine learning". The search included peer-reviewed articles published up to October 2025, without regional restrictions. Only publications available in full text were included.

We performed a comprehensive analysis of literature examining potential applications of AI in healthcare, its comparative accuracy relative to human professionals, and the anticipated ethical and practical controversies surrounding its implementation. The text underwent language polishing performed by an LLM to enhance grammatical accuracy, stylistic consistency, and overall readability.

Medical training 

The adoption of AI, particularly LLMs such as ChatGPT-4, into medical education represents a significant paradigm shift with considerable potential to optimize learning outcomes. These models can redefine curriculum development by helping educators detect content gaps, refine learning objectives, and create personalized study plans that adapt to individual student needs. LLMs also augment teaching methodologies through real-time clarifications, interactive case simulations, and virtual mentorship, facilitating deeper understanding and clinical reasoning skills [11,12]. This evolution signals a broader transformation of medical pedagogy from static, didactic instruction toward an adaptive, learner-centered framework supported by continuous data feedback. Furthermore, it underscores the growing recognition that cognitive support tools based on AI can democratize access to high-quality medical training resources, especially in institutions with limited faculty or clinical exposure opportunities.

​​AI plays an expanding role in modern medical training by strengthening diagnostic reasoning, improving feedback accuracy, and enhancing learning efficiency. LLMs such as DeepSeek-V2.5 facilitate immersive, interactive simulations that emulate real patient encounters while maintaining standardized, reproducible assessment criteria. The Adaptive Medical Training Evaluation System (AMTES), developed by Liu et al. (2025), exemplifies this advancement by integrating AI-driven virtual patients for experiential training in clinical interviewing. The study reported high reliability and accuracy, achieving human-AI consistency above 0.92 and variation below 1.2%. Importantly, AMTES provides transparent, evidence-based feedback, offering a scalable and accessible educational framework. This innovation highlights how AI complements rather than supplants human educators, enhancing both objectivity and personalization in medical education and laying the groundwork for similar integrations in clinical fields like orthopedics [13]. However, while the efficiency and precision of such systems are notable, their successful adoption depends on appropriate pedagogical alignment, faculty readiness, and ethical oversight. Without careful guidance, there is a risk that students may become overly reliant on AI-driven feedback mechanisms, potentially undermining the development of critical thinking and empathic communication skills essential to clinical practice. Nonetheless, when implemented thoughtfully, these technologies can serve as powerful cognitive amplifiers, supporting reflective learning, promoting self-assessment, and reinforcing clinical judgment within a safe, low-stakes environment such as digital case simulations, virtual patient encounters, or formative assessment platforms where learner errors do not directly impact patient care. Ultimately, the balanced coexistence of human mentorship and AI-assisted learning will likely determine the long-term value and sustainability of these innovations in medical education.

Taking medical history

A fundamental determinant in establishing accurate diagnoses is the comprehensive assessment of personal and familial medical histories. Clinical evidence suggests that medical history accounts for approximately 80% of diagnostic accuracy [14]. Clinicians must recognize that family members share not only genetic predispositions but also environmental exposures, lifestyle patterns, dietary practices, and behavioral factors. Consequently, documentation of family medical history enables healthcare providers to identify specific risk factors and hereditary susceptibilities [15]. Moreover, the process of history-taking serves as a cornerstone of the physician-patient relationship, facilitating trust, empathy, and contextual understanding that purely technological solutions may struggle to replicate. The irreplaceable human element in eliciting nuanced psychosocial information highlights the need for any automated history system to complement rather than replace clinician engagement.

Given the escalating global demand for medical consultations and increasingly challenging clinical working conditions, emerging discourse has advocated for the integration of AI in clinical history acquisition [16,17]. AI systems demonstrate considerable promise in automating medical history collection and patient triage, processes fundamental to optimizing clinical workflow and patient care. However, despite substantial research advances, these systems remain predominantly in developmental phases with limited clinical implementation. Principal barriers include challenges related to data integrity, algorithmic precision, system interoperability, and stakeholder acceptance among both patients and healthcare professionals. Incorporating perspectives from diverse stakeholders is essential for enhancing adoption rates and institutional trust. In addition, the clinical utility of such systems will depend heavily on their ability to integrate seamlessly with electronic health record platforms and to adapt to local healthcare regulations and linguistic contexts. Successful deployment will also require the establishment of transparent audit mechanisms that ensure accountability and continuous performance monitoring. From an ethical perspective, equal attention should be given to the prevention of algorithmic bias and the preservation of patient autonomy during AI-mediated information gathering. Patient autonomy may be compromised if AI systems collect, analyze, or recommend clinical actions without ensuring that patients fully understand or consent to these processes. For example, opaque algorithms could make decisions about patient care or data use that the patient is unaware of or unable to influence. Furthermore, if AI tools limit opportunities for shared decision-making by reducing clinician-patient dialogue or defaulting to automated recommendations-patients may lose control over choices that affect their health and personal information. To fully realize AI's transformative potential in healthcare, including orthopedic practice, future initiatives should prioritize rigorous clinical validation, strengthen stakeholder engagement, and address pertinent ethical and regulatory frameworks [18]. It may also be argued that sustained investment in clinician training and digital literacy will be key to bridging the human-technology divide, fostering a culture in which AI acts as an augmentative partner rather than a disruptive force within clinical diagnostics.

A pilot study conducted at a family medicine clinic within an academic medical center in Northern California revealed that the majority of participants who underwent AI-assisted interviews reported that the technology enhanced their primary care providers' understanding of their health status. Notably, most patients indicated that AI-supported tools could encourage more comprehensive disclosure of their medical histories [19]. This finding underscores that patients may perceive conversational AI as a less intimidating intermediary for sharing sensitive or embarrassing information, potentially improving the completeness and accuracy of clinical data. Nevertheless, caution is warranted: overreliance on automated systems could risk depersonalizing the consultative process and weakening the relational bond that underpins effective diagnostic practice. A balanced approach that maintains empathic communication while leveraging data-driven efficiency, therefore, remains critical to responsible AI integration in history-taking.

The study performed by Holderried et al. evaluated the use of a GPT-4-powered simulated patient chatbot for teaching medical students history-taking skills. The results show that GPT-4 provided medically plausible responses in over 99% of interactions and delivered structured feedback that closely aligned with human assessors (Cohen’s kappa = 0.832, indicating almost perfect agreement). While some feedback categories showed lower agreement due to differences in interpretation or overlapping topics, the study concludes that LLMs can effectively supplement medical education by providing realistic practice opportunities and high-quality feedback, though prompts and feedback structures should be carefully designed for optimal results [20]. Also, an LLM-based digital patient system (LLMDP) was described by Luo et al. to enhance ophthalmology history-taking skills among medical students by converting electronic health records into voice-enabled, interactive virtual patients, allowing for free-text dialogue and adaptive feedback. In a randomized controlled trial with 84 fourth-year medical students, those trained with the LLMDP system saw significantly greater improvements in medical history-taking assessment scores and demonstrated enhanced empathy compared to traditional training, with participants also reporting greater satisfaction and confidence for real patient interactions [21].

Imaging in orthopedics

Because of the strong dependence between orthopedic surgery diagnosis, as well as treatment and radiological modalities, such as computed tomography (CT), magnetic resonance imaging (MRI), and traditional radiographs, most AI and ML research focused on diagnostic imaging [6,22]. This reliance arises from the inherently visual nature of musculoskeletal pathology, where subtle morphological alterations often hold decisive diagnostic significance. Consequently, imaging provides an optimal environment for AI model development due to the availability of large, labeled datasets that facilitate supervised learning and performance benchmarking.

Langerhuizen et al.'s review reveals that AI models, particularly those employing deep learning (DL) techniques such as pretrained convolutional neural networks (CNNs), demonstrate high accuracy and near-perfect predictive ability in detecting common fractures, with area under the curve (AUC) values ranging from 0.95 to 1.0. In some studies, AI performance surpassed that of human examiners in identifying and classifying specific fracture types, such as hip and proximal humerus fractures [23]. Traditional ML models, such as Shah et al.’s method for quantifying cartilage thickness from MRI, have been effective but limited by manual feature extraction and adaptability. By contrast, DL architectures like U-Net demonstrate superior performance and efficiency in segmenting cartilage, menisci, bone, and pelvic structures, often achieving high Dice coefficients and rapid processing times. Spine segmentation and localization have benefited from multistage DL frameworks integrating CNNs, which improve accuracy in pathological conditions. Beyond segmentation, AI models show promise in automated fracture classification, matching radiologist-level accuracy across multiple fracture types. These developments underline AI’s growing role in enhancing diagnostic precision and workflow efficiency in orthopedics, although challenges remain in generalizability and clinical integration [24]. Nevertheless, despite these optimistic findings, it is important to note that many published studies are retrospective and rely on highly curated datasets, often lacking the variability encountered in real-world clinical practice. The transition from experimental validation to clinical implementation thus requires robust external testing across diverse populations and imaging hardware to ensure model reliability. Moreover, while high AUC values are encouraging, they may overstate practicality if models are not accompanied by interpretable outputs or integration frameworks that fit within radiologists’ existing workflows.

Particularly, DL with CNNs has shown significant promise in the detection of anterior cruciate ligament (ACL) tears from MRI. Several studies demonstrate that AI models can achieve diagnostic performances comparable to or exceeding those of experienced human readers by learning discriminative imaging features from large multicenter datasets. Techniques such as CNNs with customized architectures, including multi-slice inputs and region-specific image cropping, have enhanced the accuracy and sensitivity of ACL tear detection. CNN-based AI models trained on large, multicenter datasets have achieved high diagnostic performance comparable to experienced musculoskeletal radiologists. For example, one large-scale DL model trained on nearly 20,000 knee MRI scans demonstrated an AUC up to 0.939, a sensitivity of 87%, and a specificity of 91% for ACL tear detection. External validations on diverse datasets yielded AUCs of 0.922 to 0.962 with sensitivities and specificities generally above 85%, highlighting the models' robustness across populations and scanner types. Customized CNN architectures that focus on the region of interest, such as cropping images to ACL localization and using multi-slice 3D inputs, further improve accuracy. For instance, a model using a five-slice dynamic patch-based input achieved 96.7% test set accuracy, with sensitivity reaching 100% and specificity 93.3% AI methods also benefit from automated labeling through natural language processing (NLP) on radiology reports, reducing annotation costs. Notably, CNN models trained on diverse MRI datasets with different scanner types and field strengths generalize well externally, validating their potential for clinical implementation. Comparative studies between AI and human experts demonstrate AI's competitive or superior performance. A model analyzing single MRI slices showed 91% sensitivity, 86% specificity, and 88.5% accuracy, comparable to or better than some orthopedic surgeons and radiologists under test conditions. Moreover, approaches like compact parallel deep CNNs (CPDCNN) with optimized filter sizes provide efficient and accurate classification with relatively fewer trainable parameters. Overall, AI in imaging offers robust tools to assist radiologists and clinicians by improving the consistency, speed, and accuracy of ACL injury diagnosis, particularly in settings with less experienced readers or limited resources. Continued development and clinical validation of these models promise to integrate AI-assisted imaging tools into standard diagnostic workflows for musculoskeletal injuries [25-29]. However, despite strong model performance metrics, the interpretability of AI-generated results continues to pose a major barrier to clinical acceptance. The “black box” nature of CNNs can limit clinician trust, emphasizing the need for emerging explainable AI approaches that visualize decision boundaries or salient imaging features. In addition, legal accountability in cases of algorithmic misclassification remains an unresolved issue that will demand regulatory consensus before full-scale adoption can occur. Furthermore, there are inherent technical limitations in current AI technologies for medical image interpretation. These include challenges such as reduced generalizability of models to diverse patient populations and imaging settings, sensitivity to imaging artifacts and variations in acquisition protocols, and reliance on large, high-quality annotated datasets that are often scarce. Moreover, CNNs typically require substantial computational resources and advanced hardware, which can restrict deployment in low-resource clinical environments. Issues like class imbalance, small lesion detection, and the difficulty in handling multimodal data also hinder AI performance. Addressing these technical constraints alongside improving interpretability and regulatory frameworks is critical for the successful integration of AI into clinical practice.

In musculoskeletal imaging, DL techniques have been developed for tasks such as automated selection of specific MRI slices and segmentation of rotator cuff muscles with high accuracy, as demonstrated by models that achieved Dice scores around 0.93 in muscle delineation. These CNNs facilitate rapid and reliable quantification of muscle morphology, potentially enhancing clinical assessment and surgical planning. In orthopedic trauma, CNNs for fracture detection and classification have reached performance levels comparable to experienced clinicians [30,31]. Such advancements indicate that AI may soon serve not only as a diagnostic adjunct but also as a preoperative planning assistant, capable of predicting surgical complexity, estimating recovery times, and guiding personalized rehabilitation protocols. Yet, real-world evidence on the longitudinal outcomes of AI-supported orthopedic decision-making remains scarce. Future investigations should include prospective, multicenter trials that evaluate how these algorithms influence patient outcomes, surgical precision, and cost-effectiveness in routine clinical care. Ultimately, sustained collaboration between data scientists, radiologists, and orthopedic surgeons will be essential to transform current prototypical models into fully validated clinical assets.

Shen et al. checked the development and validation of a deep-learning-based diagnostic system, the AIOVFSH, designed to detect and grade osteoporotic vertebral fractures (OVFs) using plain radiographs. The system employs a multitasking network for vertebral position detection, segmentation, and fracture grading, achieving high accuracy with sensitivities around 83-84% and specificities above 94% in both internal and external validation cohorts. Compared to radiologists, the AI system showed superior diagnostic performance, particularly in reducing underdiagnosis and increasing efficiency [32].

Fracture detection

Recent advances in AI have significantly impacted the field of musculoskeletal radiology, particularly in the detection and characterization of bone fractures. Fractures represent a common diagnostic challenge in clinical practice, with missed fractures accounting for a substantial proportion of emergency department misdiagnoses and contributing to patient morbidity and physician workload [33-35]. DL-based AI models have demonstrated remarkable accuracy in fracture detection, often reaching diagnostic performance comparable to expert radiologists, orthopedic surgeons, and other physicians. For instance, recent systematic reviews and meta-analyses report pooled sensitivities and specificities above 90% for AI algorithms in fracture detection using radiographs, indicating equivalence to human experts. Moreover, commercial AI products such as OsteoDetect, FractureDetect, and BoneView have obtained regulatory clearance and shown improvements in both sensitivity and specificity when used as adjuncts in clinical workflows [33-37].

In the context of wrist fractures, AI algorithms have demonstrated superior sensitivity compared to non-specialized radiologists. A study comparing a commercially available deep neural network on wrist trauma radiographs found that AI sensitivity for fracture detection was 83%, significantly higher than the 76% sensitivity of initial radiology reports by non-expert readers. AI performance varied by anatomical region, with lower sensitivity for carpal bone fractures excluding the scaphoid, highlighting areas for further algorithm refinement. Notably, the combination of AI analysis and radiologist interpretation offered the best diagnostic accuracy [38]. For hip fractures, a systematic review and meta-analysis encompassing 39 studies with over 39,000 radiographs demonstrated that AI diagnostic models achieved a mean sensitivity of 89.3% and specificity of 87.5%, comparable to expert clinicians. The odds ratio for diagnostic error did not differ significantly between AI and human readers. Moreover, AI-based models predicting postoperative outcomes showed performance similar to traditional statistical models, indicating that while AI automated diagnosis is promising, its benefit for outcome prediction may be limited by current model interpretability and complexity [39]. A comprehensive systematic review and meta-analysis of fracture detection across radiographs and CT scans confirmed that AI and clinicians have comparable diagnostic performance, with pooled sensitivity and specificity around 91-92% [40]. In pediatric fracture assessment, recent systematic reviews reveal rapid growth in AI model development with reported diagnostic accuracies ranging from 85% to 100%. AI assistance improved human reader performance, particularly among less experienced radiologists (under five years of clinical experience), suggesting AI's potential role as a diagnostic aid in settings where pediatric expertise is scarce [41,42]. 

Diagnosis

The use of AI in diagnostic medicine, particularly NLP models like ChatGPT, shows promising yet inconsistent potential for medical self-diagnosis [43]. Fukuzawa et al.'s study showed that when AI is provided with comprehensive clinical data, including medical history and present physical findings, it can achieve 93.3% accuracy in diagnosis [14]. Despite such encouraging metrics, diagnostic dependability varies substantially depending on the input data’s completeness, phrasing, and contextual specificity. Unlike structured datasets used in clinical trials, patient-provided input is often vague, subjective, or incomplete, which significantly reduces algorithmic accuracy in real-world applications. This discrepancy underscores the need for standardized input protocols or hybrid systems where AI operates as an initial triage assistant rather than an independent diagnostic authority.

ChatGPT has been evaluated for its diagnostic performance across several common orthopedic disorders, such as carpal tunnel syndrome, lumbar spinal stenosis, and osteoarthritis. Its precision tends to decrease as the complexity of the disease increases, resulting in lower diagnostic accuracy for more complex orthopedic conditions. Although the model achieves high diagnostic concordance for certain well-defined conditions, it exhibits reduced reliability in disorders characterized by multifocal or overlapping symptomatology, such as cervical myelopathy [43]. These findings emphasize that while generative models excel at pattern recognition and probabilistic reasoning, they remain constrained by the absence of direct sensory data, such as physical examination cues, which are critical in orthopedic evaluation. Moreover, the model’s outputs may reflect training-data biases or an overrepresentation of Western clinical knowledge bases, potentially affecting diagnostic generalizability across diverse patient populations. From a clinical governance perspective, overconfidence in AI-produced diagnostic suggestions could inadvertently promote patient misinterpretation of symptoms and delay appropriate medical consultation. Nonetheless, when used responsibly under professional oversight, ChatGPT-like systems could enhance patient education, facilitate pre-consultation data capture, and streamline initial triage processes, freeing clinicians to focus on complex analytical tasks.

Evaluating AI potential in supporting learning by academic tests

A synthetic way of assessing LLMs' abilities may be measuring their effectiveness in academic tests. Vaishya et al. conducted a study comparing ChatGPT 3.5, ChatGPT 4.0, and Bard Google's accuracy in orthopaedic postgraduation exam questions, which showed significant differences between these models. Bard Google answered all questions correctly, while ChatGPT 3.5 and 4.0 achieved, respectively, 45% and 54.2% of correct answers. The differences may be caused by Bard Google having real-time data access through Google, providing the most up-to-date information [44]. However, such comparisons should be interpreted with caution, as live web access grants Bard a substantial informational advantage that cannot be equated with true reasoning capability or domain comprehension. This distinction underscores the importance of differentiating between data retrieval capacity and genuine analytical understanding when evaluating AI systems in academic contexts. 

ChatGPT 4.0 demonstrated performance exceeding 50% accuracy on orthopedic assessment tasks, and also exhibited competence in radiological domains. A study has shown that ChatGPT 4.0, when asked questions from the Fellowship of the Royal College of Radiologists, was able to answer correctly. ChatGPT 4.0 correctly answered 74.8% of the Part 1 true/false questions, narrowly missing the Spring 2023 passing threshold of 75.5%.In the Part 2A examination, ChatGPT-3.5 achieved a score of 50.8% on single best answer (SBA) questions, whereas GPT-4 scored significantly higher at 74.2%. Given that the Winter 2022 Part 2A pass mark was 63.3%, GPT-4 comfortably surpassed the required standard [45]. These results mark a notable milestone in LLM progression, illustrating measurable academic growth across model generations. Nevertheless, the proximity of GPT-4’s scores to passing thresholds also highlights its inconsistent performance across question types, suggesting that while the model excels in pattern recognition and factual knowledge synthesis, it continues to exhibit limitations in higher-order critical reasoning.

LLMs such as ChatGPT, Bard, and Bing Chat have shown performance in orthopaedic board-style examinations that is comparable to first-year orthopaedic surgery residents. Their diagnostic capabilities and their ability to generate clinically relevant answers were systematically evaluated using Orthopaedic In-Training Examination (OITE) question banks, enabling direct comparison with both medical students and residents at various stages of training. Importantly, these AI tools proved capable of assimilating textual medical information and applying clinical guidelines with reasonable accuracy, despite the absence of multimedia content typically accessible to human examinees [45]. Recent advancements in LLM AI, particularly ChatGPT-4.0 and a custom-trained model known as Orthopod, have demonstrated the capacity to answer standardized board-style questions in orthopaedic surgery at a high level of proficiency. In a comparative study using the 2022 OITE practice questions, both ChatGPT-4.0 and Orthopod exhibited accuracy rates exceeding 70%, with no statistically significant difference in their overall performance or in subspecialty categories. Both models were capable of providing well-reasoned responses and detailed explanations to multiple-choice questions, revealing their potential as educational tools in orthopaedic resident training [46]. GPT-4 consistently cited reliable sources, predominantly journal articles with significant impact factors, reinforcing its utility as an educational aid [47].

However, not all studies have shown similar results. The one performed by Lum evaluated ChatGPT's performance on the Orthopaedic In-Training Examination (OITE), revealing that the model correctly answered approximately 48% of text-based multiple-choice questions, corresponding to the knowledge level of a first-year orthopaedic resident. The model's performance declined with increasing question complexity, particularly in tasks requiring application and synthesis of knowledge, indicating limitations in its ability to transfer factual information to clinical problem-solving. Notably, ChatGPT’s performance fell below the passing threshold for the American Board of Orthopaedic Surgery written examination, especially compared to more advanced residents [48]. These findings reaffirm the model’s apparent ceiling effect when faced with tasks requiring nonlinear reasoning, real-time judgment, or interpretation of ambiguous data. For instance, the model struggles when it must dynamically reprioritize a differential diagnosis as new, conflicting information appears in the chart, or when subtle exam findings point toward a rare complication rather than the statistically most common cause. It also performs inconsistently when asked to balance guideline-based recommendations with individual patient constraints, such as comorbidities, social factors, or patient preferences that are only implicitly stated in the record. These limitations reveal that despite rapid algorithmic advancement, current LLMs are not yet capable of replicating the nuanced, context-driven reasoning expected of a competent clinician.

Despite these limitations, the model demonstrated potential as a supplementary educational tool by efficiently handling recall and interpretation tasks and showing adaptability when provided with corrective feedback [8,49]. In this capacity, AI-driven LLMs may assume a formative role in medical education, serving as interactive tutors that adapt to learners’ cognitive patterns, reinforce factual retention, and simulate clinical reasoning under supervision. However, their responsible incorporation into curricular design will require structured guidance, evaluation frameworks, and ethical oversight to ensure that AI complements rather than substitutes rigorous clinical education. Ultimately, the integration of LLMs into academic medicine should prioritize transparency, educational value, and alignment with established learning outcomes rather than mere exam performance. Table 1 compares studies evaluating particular LLMs.

**Table 1:** Comparison of studies evaluating particular large language models MCQ: multiple-choice question, LLM: large language model, PGY-1: postgraduate year 1, OITE: orthopaedic in-training examination

Authors	Year	Compared models	Summary
Vaishya et al. [44]	2024	ChatGPT-3.5, ChatGPT-4, Bard (Gemini)	ChatGPT-4 (54.2%) outperformed ChatGPT-3.5 (45%); Bard achieved 100% accuracy on MCQs. Only Bard surpassed typical exam passing thresholds.
Guerra et al. [45]	2025	ChatGPT-3.5, Bing Chat, Bard	All LLMs (ChatGPT-3.5: 46.3%, Bard: 51.4%, Bing: 52.4%) performed similarly to PGY-1 residents and were well below passing benchmarks for senior residents.
Magruder et al. [46]	2025	ChatGPT-4, Orthopod (custom GPT-4)	ChatGPT-4 (73.4%) and Orthopod (71.0%) answered OITE exam questions with high and statistically similar accuracy, exceeding most reported LLM studies, with no significant difference found.
Kung et al. [47]	2023	ChatGPT-3.5, GPT-4	GPT-4 (73.6%) scored above the passing level for board exams and outperformed ChatGPT-3.5 (54.3%), indicating rapid improvements in LLM capabilities
Lum [48]	2023	ChatGPT (version unspecified)	ChatGPT correctly answered 47% of written orthopaedic board questions, matching PGY-1 level but far below passing for board exams. Performance declined with higher cognitive demand questions.

Concerns

Concerns about the implementation of AI in healthcare revolve around ethical, technical, and operational challenges that complicate its safe, equitable, and effective integration into clinical practice. Core issues include the risk of perpetuating biases present in training data, which can exacerbate health inequities among marginalized populations. The opacity of AI decision-making, often described as "black box" models, undermines explainability and trust, making it difficult for clinicians to interpret AI recommendations reliably. These concerns are not merely theoretical; empirical studies show that biased algorithms have already produced uneven diagnostic and predictive outcomes in conditions such as cardiovascular disease and dermatologic cancers, disproportionately affecting underrepresented demographic groups. Consequently, building fairness-aware models with traceable decision pathways is increasingly recognized as a prerequisite for ethical AI deployment in healthcare.

Furthermore, accountability remains ambiguous when AI errors occur, raising questions about legal liability among developers, healthcare providers, and regulators. Data protection and cybersecurity represent further areas of concern, especially given the sensitive nature of medical information and potential vulnerabilities to data breaches and malicious attacks. In an era of interconnected health information systems, breaches could have cascading impacts, leading not only to privacy violations but also to disruptions in clinical continuity and patient trust. Establishing clear standards for data stewardship, informed consent, and cybersecurity auditing is therefore essential. From a legal standpoint, there is growing consensus that governance frameworks should delineate differentiated responsibilities among AI designers, institutional adopters, and end users, ensuring accountability proportional to each actor’s role in the decision chain.

Effectively addressing these multidimensional challenges demands robust validation protocols, transparent methodological disclosure, ethically grounded oversight, and collaborative frameworks for human-AI integration, ensuring that the promise of AI in healthcare is realized safely [7,50-54]. Equally important is the cultivation of digital ethics literacy among clinicians, enabling them to critically appraise AI recommendations and to recognize potential algorithmic limitations in real time. Without this competence, even the most transparent and validated systems may fail to achieve meaningful clinical acceptance. Ultimately, the success of AI deployment in healthcare will depend not only on technological excellence but also on nurturing a culture of ethical reflexivity, interdisciplinary dialogue, and sustained regulatory vigilance.

Discussion

The integration of AI into medical education and clinical practice represents a paradigm shift with profound implications for both pedagogy and patient care. LLMs such as ChatGPT-4 and advanced DL systems have demonstrated the capacity to identify knowledge gaps, personalize curricula, and simulate complex clinical scenarios, contributing meaningfully to more effective and adaptive learning environments. These systems facilitate interactive learning through tools like real-time case simulations, virtual mentorship, and dynamic feedback, thereby supporting the development of clinical reasoning skills and fostering deeper understanding among medical trainees. Empirical evidence suggests that AI-powered evaluation platforms can reliably assess student performance, offering objective, reproducible feedback that supports both learners and educators. Importantly, the use of AI-driven conversational agents in history-taking tasks has been shown to encourage comprehensive disclosure from patients, potentially enhancing diagnostic accuracy and building more complete clinical records.

However, the promise of AI must be balanced with a critical evaluation of its limitations and risks. A major concern lies in the potential for AI systems to perpetuate existing biases embedded in training data, potentially exacerbating disparities in healthcare outcomes among diverse populations. Moreover, the "black box" nature of DL models poses interpretability challenges, making it difficult for clinicians to fully trust or understand algorithmic recommendations. Legal uncertainties regarding accountability for AI-driven errors, as well as robust requirements for data protection and cybersecurity, further complicate the landscape. While AI models may excel in processing large, curated datasets and achieving high diagnostic accuracy, their clinical reliability may be limited by the variability inherent in real-world practice. Overreliance on automated feedback systems could also risk undermining critical thinking and empathy, the hallmarks of high-quality medical care, by deprioritizing the irreplaceable value of human judgment and patient interaction.

Effective and ethical adoption of AI in medicine demands rigorous clinical validation, stakeholder engagement, and continual oversight [55,56]. Transparent audit mechanisms and interdisciplinary collaboration are essential to building trust and ensuring seamless integration with electronic health record systems. In addition, sustained investment in digital literacy and ethical training for clinicians is crucial for fostering the critical reflexivity needed to assess AI-generated insights responsibly. Ultimately, the long-term value of AI in healthcare will depend on its ability to function as a cognitive amplifier, complementing, rather than substituting, the diverse expertise and empathetic presence of medical professionals. This balanced, ethically guided integration will lay the foundation for a truly adaptive and equitable future in both medical education and clinical practice.

## Conclusions

The integration of AI that holds the capacity to transform medical research by facilitating the rapid and precise analysis of extensive datasets, which include demographic, genetic, clinical, and surgical information derived from thousands of patients in medical imaging, has increasingly transformed orthopedic practice by enhancing diagnostic accuracy, optimizing imaging modality selection, and facilitating clinical decision-making processes. AI systems, such as those based on NLP models like ChatGPT, assist clinicians by synthesizing patient data, medical histories, and radiological findings to suggest appropriate imaging techniques and support differential diagnoses. Furthermore, AI contributes to personalized treatment planning through predictive analytics and data-driven insights derived from extensive imaging datasets. Moreover, AI-driven image recognition assists in identifying anatomical structures and guiding instrument placement during complex surgeries such as joint replacement.

Despite these advancements, challenges remain in the accuracy and reliability of AI-generated information, potential biases in training data, ethical and legal considerations, and the model’s current inability to replace expert clinical judgment in complex or nuanced cases. Other barriers to overcome are the skepticism of professionals, adaptation to digital care, or troubles with the integration of systems. Continued development and validation of AI tools in medical imaging are vital to fully realize their potential in improving patient outcomes and clinical workflow efficiency in orthopedics.
